# Evidence-based medicine – an appropriate tool for evidence-based health policy? A case study from Norway

**DOI:** 10.1186/s12961-016-0088-1

**Published:** 2016-03-05

**Authors:** Kirsti Malterud, Anne Karen Bjelland, Kari Tove Elvbakken

**Affiliations:** Research Unit for General Practice, Uni Research Health, Kalfarveien 31, N-5032 Bergen, Norway; Department of Global Public Health and Primary Care, University of Bergen, Bergen, Norway; The Research Unit for General Practice and Section of General Practice, Department of Public Health, University of Copenhagen, Copenhagen, Denmark; Department of Social Anthropology, University of Bergen, Bergen, Norway; Department of Administration and Organization Theory, University of Bergen, Bergen, Norway

**Keywords:** Case study, Evidence-based medicine, Health policy, Knowledge bases, Systematic reviews

## Abstract

**Background:**

Evidence-based policy (EBP), a concept modelled on the principles of evidence-based medicine (EBM), is widely used in different areas of policymaking. Systematic reviews (SRs) with meta-analyses gradually became the methods of choice for synthesizing research evidence about interventions and judgements about quality of evidence and strength of recommendations. Critics have argued that the relation between research evidence and service policies is weak, and that the notion of EBP rests on a misunderstanding of policy processes. Having explored EBM standards and knowledge requirements for health policy decision-making, we present an empirical point of departure for discussing the relationship between EBM and EBP.

**Methods:**

In a case study exploring the Norwegian Knowledge Centre for the Health Services (NOKC), an independent government unit, we first searched for information about the background and development of the NOKC to establish a research context. We then identified, selected and organized official NOKC publications as an empirical sample of typical top-of-the-line knowledge delivery adhering to EBM standards. Finally, we explored conclusions in this type of publication, specifically addressing their potential as policy decision tools.

**Results:**

From a total sample of 151 SRs published by the NOKC in the period 2004–2013, a purposive subsample from 2012 (14 publications) advised major caution about their conclusions because of the quality or relevance of the underlying documentation. Although the case study did not include a systematic investigation of uptake and policy consequences, SRs were found to be inappropriate as universal tools for health policy decision-making.

**Conclusions:**

The case study demonstrates that EBM is not necessarily suited to knowledge provision for every kind of policy decision-making. Our analysis raises the question of whether the evidence-based movement, represented here by an independent government organization, undertakes too broad a range of commissions using strategies that seem too confined. Policymaking in healthcare should be based on relevant and transparent knowledge, taking due account of the context of the intervention. However, we do not share the belief that the complex and messy nature of policy processes in general is compatible with the standards of EBM.

## Background

Knowledge is an essential foundation for informed decisions in healthcare policy, and there is broad agreement that best available research evidence should be used [1-3]. However, what constitutes best available research evidence for this purpose? In combination with the vast amount of available information, an increasing expectation that decisions should be transparent to be explicitly justified has led to the notion of evidence-based policy (EBP), modelled on the principles of evidence-based medicine (EBM) [1,2]. This article explores the relationship between these two concepts.

In 1971, the Scottish epidemiologist Archie Cochrane (1909–1988) [4,5] raised concerns about effectiveness, efficiency and equality in healthcare, calling for randomized control trials (RCT) as the gold standard of evidence [4]. His ideas were endorsed by the Canadian epidemiologist David Sackett (1934–2015), who encouraged careful evaluation of health interventions to improve the effectiveness of medical care [5]. From this background emerged the concept of EBM, defined as “*the conscientious, explicit, and judicious use of current best evidence in making decisions about the care of individual patients*” [6]. The aim was to close the gap between evidence and practice, making it possible to evaluate health services on the basis of scientific evidence rather than on clinical impression, anecdotal experience, ‘expert’ opinion or tradition [7,8].

### Systematic reviews (SRs)

SRs with meta-analyses gradually became the methods of choice for synthesizing research evidence, with further development of systematic approaches for judging quality of evidence and strength of recommendations beyond intervention studies [7,9-11]. Standard procedures for SRs of health interventions are presented in the PRISMA Statement [12-14], in which transparency with regard to search strategy, inclusion criteria and data extraction is considered crucial. The SR should therefore contain explicit statements of questions addressed with reference to participants, interventions, comparisons, outcomes and study design (PICOS). The quality of evidence and the strength of recommendations are systematically assessed using the Grading of Recommendations Assessment, Development and Evaluation (GRADE) system [9].

EBM and a broad range of SRs formed the basis for development and implementation of various guidelines for clinical practice [8]. EBM had also been established to provide evidence for health policy decision-making. In Great Britain, Tony Blair’s Labour government emphasized rigorous scientific analysis as a knowledge base for improved policymaking, and a white paper from 1999 advanced the concept of EBP [15]. According to EBP, research evidence should meet the requirements of the EBM principles and hierarchy of evidence preferring RCTs in support of health policy [1,4,8,16]. However, transfer of the ‘evidence based’ concept from clinical practice to health policy has not been straightforward [1]. Critics have, for example, argued that the relation between research evidence and service policies is weak [3] and that the notion of EBP rests on a gross misunderstanding of policy processes [2]. While some policy decisions are simple and lucid, others are complex or incoherent. We therefore sought to explore EBM standards and knowledge requirements for health policy decision-making. In this article, we present an empirical basis for such discussions, focused on the relationship between EBM and EBP.

In Norway, the links between EBM and EBP were institutionalized in 2004 with the establishment of the Norwegian Knowledge Centre for the Health Services (NOKC). The details of the process are described in an anniversary monograph [17]. This organization presents a unique opportunity to study EBM as operated by a government unit whose knowledge deliveries are intended to enhance health policy decision-making. We conducted a case study of the NOKC as a point of departure for our research.

## Methods

To begin, we will describe our overall research design and strategy [18], followed by a description of the study context. Finally, in this section, we present our procedures for the systematic collection and analysis of empirical data.

### Research design and strategy

The case study was conducted in three phases. First, we searched for information describing the background, tasks, organizational framework, establishment, values and development of the NOKC to define the study context. In the second phase, we identified, organized and selected official publications from the NOKC, establishing an empirical sample of typical top-of-the-line knowledge deliveries adhering to EBM principles and standards. In the third phase, we explored the conclusions drawn in this type of publication, specifically addressing their potential as policy decision-making tools. Our analysis provided arguments for a critical discussion of EBP and its underlying assumptions.

### Study context: the NOKC

In Norway, adoption of EBM was officially enacted in 2004 with the establishment of the NOKC, an independent government unit administered by the Norwegian Directorate of Health [19]. The establishment of the NOKC was based on “*a need to enhance the underpinning of decisions in ministries, directorate and regional health enterprises to improve achievement of aims within health policy and encounter future professional challenges*” [19] (our translation, p. 98). Resources were assembled by merging three existing health service research units outside the universities, including a group of researchers who established the Norwegian branch of the Nordic Cochrane Centre. The initial annual budget of the NOKC in 2004 was NOK 33.8 million, increasing to NOK 182 million in 2014 [20]. Reviewing the available documentation, we have compared the priority aims and practices of the NOKC to established EBM standards [4,6,8,21-24]. We also particularly searched for authoritative demonstrations of epistemological positions and institutional preferences with regard to knowledge deliveries.

According to the anniversary monograph, the NOKC’s two core activities in its first decade were (1) knowledge synthesis (different types of SRs) and (2) monitoring (quality assessments, user satisfaction surveys) [17]. Other activities of the NOKC include strategies for methodological innovation and implementation of EBM in developing countries [25,26]. In the present study, we focused on activities related to knowledge synthesis, which was estimated to account for 25% of the institution’s total person hours in 2012 [17] (p. 176). NOKC strategies for knowledge synthesis have been explicitly drawn from EBM, with reference to methods from the Cochrane Collaboration [27]. In addition, the NOKC website exhibits a broader conceptual perspective, promoting an ideology of knowledge-based practice as a balance between research evidence, experience-based knowledge and patient values and preferences [28].

### Collection and analysis of empirical data: selected publications from NOKC 2004–2013

In October 2014, we conducted a systematic examination of all publications presented on NOKC’s website, reflecting the organization’s tasks, priorities and knowledge deliveries. SRs complying with EBM standards were the highest-ranked delivery format [9,11,29].

To identify SRs from NOKC’s website, we applied the organization’s own taxonomy of publication types. From our searches, it became clear that publications other than those classified as SR also contained elements of SR methodology. This overlap with well-defined SRs was noticed in publications classified as health technology assessments, brief reviews and reviews with sorted literature. The most consistent EBM-compatible methodology was identified in publications classified by NOKC as SRs. Publications in this category from the period 2004–2013 were therefore chosen as a purposive sample from which to explore links and relations between EBM and EBP.

The sample was organized to reflect the number and type of NOKC-produced SRs, contextualized in terms of total output during these years. As the NOKC webpages offered no consistent thematic classification of SRs, we developed four categories to provide a rough overview of health areas covered by these publications: (1) screening/diagnosis; (2) treatment/medication/vaccination; (3) prevention/psychosocial interventions; and (4) initiatives within health and social services. In March 2015, we noted that the NOKC had totally revised their website, including their classification of publication types. While we found complete agreement for lists of SRs for the period 2007–2013, our lists for the years 2004–2006 had to be adjusted to reflect the new NOKC scheme. While still providing relevant information for our purposes, we concluded that the quantitative accuracy of this approach to data collection would be moderate, while still providing relevant information for our purpose. However, it was no longer possible to sort the full range of publications by type, as the original taxonomy had not been consistently applied by the NOKC.

We then conducted a formative qualitative evaluation [30] of SRs from a random recent year (2012). More specifically, we reviewed the conclusions of this subsample of reports to explore their potential as evidence to support informed healthcare policy decision-making. Drawing on perspectives from the rhetorics of health and medicine, we assessed the persuasive power of the conclusions mediated by the language used, especially with regard to terms indicating positions of certainty or reluctance [31]. This process was conducted by systematic negotiation between the authors in pursuit of consensus.

The summary of quantitative results below displays the number, proportion and distribution (annual and thematic) of SRs from the NOKC. We then report our findings on the potential of these SRs in support of health policy decision-making.

## Results

**Fig. 1:**
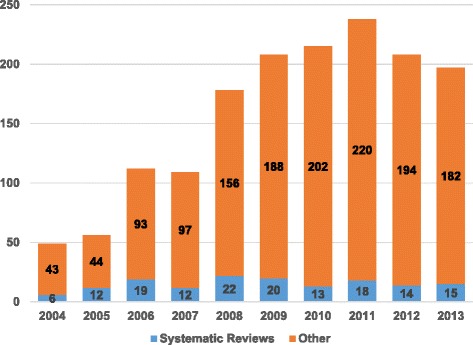
**Annual number of publications from the Norwegian Knowledge Centre for the Health Services (2004–2013).**

During the period 2004–2013, the NOKC produced 1,570 publications in total, including SRs, systematic literature searches, sorted brief reviews, reports from the Knowledge Centre, reports from GRUK (quality improvement), reports from PasOpp (patient experiences), memorandums, health technology assessments and other published material (Fig. 1). During that decade, a total of 151 SRs were published (annual range 6–22, median 14.5), representing 6–21% of NOKC’s annual output. The highest proportion of these came from the institution’s first years of activity (2005 and 2006); since that time, the level has remained stable.

**Fig. 2:**
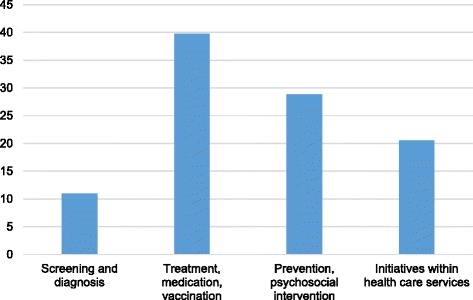
**Themes covered by systematic reviews (total numbers) from the Norwegian Knowledge Centre for the Health Services (2004–2013).**

The thematic distribution of SRs is shown in Fig. 2. The majority of SRs were related to treatment/medication/vaccination, followed by prevention/psychosocial interventions, advances in health and social services and screening/diagnosis.

**Fig. 3:**
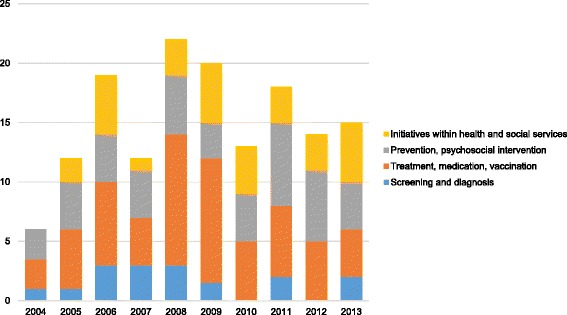
**Systematic reviews (annual number and distribution of themes) from the Norwegian Knowledge Centre for the Health Services (2004–2013).**

Based on our review, 2012 was found to be a fairly typical year (Fig. 3).

During 2012, the NOKC published 14 SRs. Within this sample, six SRs dealt with prevention/psychosocial interventions, five with treatment/medication/vaccination and three with advances in health and social services. None of that year’s SRs dealt with screening/diagnosis. Commissions came from government directorates (n = 6), trade unions for health professionals (n = 4), hospitals (n = 3) and the Norwegian Cancer Society (n = 1).

Altogether, 57,368 studies were screened to produce a total of 351 studies for synthesis in 2012. The average number of initial records identified for each SR was 4,098 (range 263–10,188), and the average number of included studies for each SR was 25 (range 3–91). While most of the included studies were SRs, some used RCTs or observational studies as their point of departure (Table 1). In this sample of 14 SRs, we observed that the major rhetorical pattern in the conclusions advocated caution in various forms. In some reviews, such as one relating to list size and quality of care among general practitioners, no relevant effect studies were identified. In a majority of SRs, some documentation had been retrieved, but this consisted predominantly of studies, often few, small or old, with heterogeneity of intervention types. Terms like ‘moderate’, ‘low’ or ‘very low quality’ occurred frequently with reference to the available documentation. These classifications seem to be drawn from standard ratings of evidence quality [9]. Several of the conclusions strongly recommend caution regarding interpretation because of risk of bias, indicating the possibility of systematic skewness and low validity of results. While some of the SRs stated that no certain conclusions could be drawn, others employed a striking level of cautious terminology in presenting their conclusions, such as ‘probably increases’, ‘possibly increases’, ‘increases perhaps’, ‘may reduce’, ‘uncertain’, ‘difficult to conclude’. This terminology apparently reflected the rating system.

**Table 1:** Systematic reviews (themes, titles, total hits and included studies) from the Norwegian Knowledge Centre for the Health Services (2012)

Theme	Number	Titles of reports	Initial records	Included studies
Screening/Diagnosis		None		
Treatment/Medication/Vaccination	18-2012	Psychological treatments for non-specific pain	4,300	6 SR
	17-2012	Effect of multidisciplinary, team-based rehabilitation, including education, in rheumatoid arthritis	1,441	11
	13-2012	Effect of long-term mechanical ventilation (LTMV) part 1 – neuromuscular disease or central respiratory failure	5,442	34 (1 SR, 3 RCT)
	14-2012	Effects of LTMV part 2 – thoracic restrictive disorders or obesity hypoventilatory syndrome	6,978	33 (2 RCT)
	02-2012	The effects of sexual therapy interventions for sexual problems	2,805	43 (9 SR, 34 RCT)
Prevention/Psychosocial interventions	12-2012	Effects of organised follow-up of behaviour that may increase risk of disease in adults	10,188	23
	08-2012	Effect of interventions to ease transitions for children and adolescents with disabilities	263	3 SR
	07-2012	The effectiveness of primary interventions to prevent the use of tobacco, alcohol and other drugs among children and adolescents	4,518	10 SR
	06-2012	The effectiveness of health promotion and preventive interventions on nutrition, physical activity, obesity, and sexual health in children and adolescents	4,518	6 SR
	10-2012	Effects of support and follow-up interventions for people with severe mental illness	2,674	17 SR
	03-2012	Interventions for tobacco control in low- and middle-income countries: evidence from randomised and quasi-randomised studies	2,806	45 (26 RCT)
Initiatives within health and social services	05-2012	Effects of organisational interventions for mental health services	4,258	17 SR
	01-2012	List size and quality of care among general practitioners within the Regular General Practitioner Scheme	3,806	0/91^a^
	09-2012	Interventions for reducing seclusion and restraint in mental health for adults	3,361	12

^a^ No effect studies, 91 observational studies

SR, Systematic reviews; RCT, Randomized controlled trials

Only one of these 14 SRs from 2012 (dealing with interventions to prevent use of tobacco, alcohol and drugs among children and adolescents (07-2012)) concluded that extensive and high quality documentation had been identified. While the report identified a range of effective interventions, the language of caution again appeared (e.g. ‘possibly effective’, ‘likely not effective’). Another related SR, dealing with interventions regarding nutrition, physical activity, obesity and sexual health in children and adolescents (06-2012), reported that substantial documentation allowed the authors to draw some conclusions. Nevertheless, they expressed some reservations about the broad scope of the documentation, which meant that the recommended interventions were at a rather general level. For the remaining 12 SRs, the authors’ conclusions are characterized in every case by an overarching caution.

## Discussion

A major task for the NOKC as an independent government organization is to provide knowledge that will underpin health policy decisions [19]. Institutional strategies are clearly influenced by EBM values and methods [17], installing SRs as the most valued format for knowledge delivery [32]. From a total sample of 151 SRs published by the NOKC in the period 2004–2013, our purposive subsample served to demonstrate that most of these reports advised major caution in relation to their conclusions owing to the quality or relevance of the underlying documentation. Although our case study did not include a systematic investigation of uptake and policy consequences, it became apparent that SRs are not appropriate universal tools for health policy decisions. Below, we discuss the impact of these findings.

### EBM in clinical decision-making

EBM was developed as a strategy to inform patient care as well as health policy decisions. We are not the first to suggest that the evidence hierarchy of EBM, which gives precedence to quantifiable and supposedly universal knowledge, may be too confined to meet the needs of clinical decision-making in respect of the individual patient [33,34]. Resistance to this confined conception of evidence was expressed by members of clinical disciplines where experientially based, tacit knowledge is required, as distinct from the formal knowledge offered by science [35]. Similar arguments regarding the nature of knowledge had previously been articulated from general practice researchers, with regard to the limitations of universal knowledge and the impact of particular and individualized knowledge in clinical practice [33,36-40]. Approaching clinicians, the NOKC has invested considerable resources in implementation, with courses, websites and organized access to research literature for healthcare professionals. The centre has conceptualized a platform for knowledge-based practice in which mainstream EBM is integrated with the experience-based and user-based knowledge in a local context [17].

Pope described EBM as a contemporary social movement with a high profile, although not overly successful [35]. After a decade of operation, the values of the EBM movement and the corresponding evidence hierarchy are still embraced by the NOKC [17]. The original idea of integrating individual clinical expertise with best evidence [6] seems to have gradually vanished in the shadow of meta-analyses, RCTs and algorithmic rules [34,41]. Procedures for the assessment and synthesis of qualitative evidence have recently been included among EBM standards and tools by the Cochrane Collaboration [42], but such approaches were not prominent in the sample of SRs we explored.

We find it plausible that EBM principles applied in SRs that synthesize current research knowledge in a standardized and transparent format may well enhance clinical decision-making. However, the tool seems most appropriate and capable of providing a clear answer when the research question fits the hierarchy of evidence and the PICO formula [9,12]. A typical example would be whether medication X is a better treatment than medication Y for patients suffering from a single and well-defined disease, as in the prevention of eye infection after cataract surgery [43]. Another example of the tool’s appropriateness would be to ask whether test Z as applied to a defined population will have an impact on morbidity or mortality (disease-specific or all-cause, respectively) for tests and conditions such as prostate-specific antigen and prostate cancer [44].

In clinical practice, however, such clear-cut decision spaces rarely exist, especially in the primary healthcare setting, where multimorbidity is commonplace [45]. This is probably one important reason for the limited compliance among general practitioners to EBM-based guidelines [46]. The challenges of EBM-based clinical decision-making become more apparent in complex and behavioural interventions in more heterogeneous populations, as in most of the sample we examined.

### EBM as a tool to guide health policy decisions

Our exploration of EBM indicates its shortcomings as a tool for clinical practice, in that its universal and standardized evidence base fails to address the particular question and context for decision-making. These concerns are no less valid in assessing EBM as a tool for policy decision-making (even though the current Cochrane terminology refers to evidence-informed health decision-making) [47]. Dobrow et al. [48] emphasize current conceptual deficiencies and the limited attempts to acknowledge the role of context in evidence-based decisions. To understand the complex adaptive nature of health systems, the context, acceptability and feasibility of interventions must be highlighted [49]. Mixed-methods approaches have been proposed as a means of studying complex health systems at patient, provider, institutional and system levels [26,42,50,51]. To date, however, our sample of SRs from 2012 indicates little attention to contextual or qualitative matters in NOKC knowledge deliveries.

Even with better research studies and more solid conclusions than those of our sample from 2012, policy decision-making processes demand much more than knowledge translation, defined by WHO as “*the synthesis, exchange and application of knowledge by relevant stakeholders to accelerate the benefits of global and local innovation in strengthening health systems and advancing people’s health*” [52]. This conception represents the ‘know-do’ gap to be bridged between scientific facts and policymaking as a simple pipeline model, in which incoming evidence underpins decisions [53,54]. Our analysis of the available empirical data suggests that, if such a pipeline were operating, the flow of conclusions and corresponding policy decisions would be very low.

Knowledge deliveries that repeatedly communicate a broad lack of quality evidence across most tasks may indicate the inadequacy of standard tools for a government unit commissioned to enhance decision-making. One hypothesis arising from our empirical analysis is that the NOKC (and, probably, comparable institutions within the EBM movement) addresses too broad a range of inquiries, using too confined a range of tools. The main problem with the EBM approach is its restricted and simplistic approach to scientific knowledge [55]. The standardizing imperative that underpins EBM strategies is neither compatible with all questions of relevance for policymaking nor with scientific reasoning. It would be interesting to look more closely at the decision-making processes and the role of the authorities in how assignments are accepted, modified or dismissed. Knowledge is always situated [56]. The NOKC SRs are no exception, informing both the question asked and the consequences of the answer.

In a critical analysis of evidence-based policy research, Oliver et al. [54] concluded that many approaches within this area are naïve in neglecting how policy is influenced and constituted. They point to a loss of clarity about what constitutes and defines ‘evidence’ and ‘policy’, neglecting “*the policy process itself as a contested area of negotiation*” and “*the messy, complex, and serendipitous nature of policymaking*” [54]. Comparing three SRs on EBP research, Oliver et al. [54] argue that, without an understanding of the complex processes of policy and knowledge mobilization, researchers who make policy and practice recommendations will simply be ignored. Orton et al.’s [1] SR of the use of research evidence in public health decision-making processes also concluded that the impact of research evidence was often indirect and had to compete with other influences.

We believe that current EBM-based NOKC studies seek to provide a legitimate basis for health policy decision-making in Norway, and we endorse the idea that certain domains of inquiry may be well suited to such approaches. However, given the large proportion of recent NOKC deliveries that lack substantial conclusions, we must also reflect on the potential policy consequences of ‘empty’ knowledge deliveries. As a broad range of complex interventions cannot be adequately evaluated by the EBM format, there will be limited availability of ‘authorized’ research documentation, and SRs will be unable to offer strong conclusions about positive effects. This is not the same as documentation of negative or zero intervention effects. Often, the issues leading to SR commissions are controversial, and the delivery is expected to recommend a direction or choice. The present findings highlight the possibility of policy decision-making in which political interests (as in “this government does not want to support such a reform”) may be legitimized by SRs that draw weak or deficient conclusions, which are then interpreted as evidence-based warnings against the intervention in question. In this way, the EBM evidence hierarchy may actually contribute to concealing the foundation of policy decision-making rather than providing transparency.

## Conclusions

In this case study exploring selected publications from the NOKC, we have demonstrated that EBM is not universally suited to knowledge provision for every kind of health policy decision-making. Our analysis raises the question of whether the EBM, represented here by an independent government organization, undertakes too broad a range of commissions using a range of strategies that seem too confined. As far as possible, healthcare policymaking should be based on relevant and transparent knowledge, taking account of the context of the intervention. However, we do not share a belief that the complex and messy nature of policy processes in general is compatible with the standards of EBM.

## Abbreviations

EBM: Evidence-based medicine
EBP: Evidence-based policy
NOKC: Norwegian Knowledge Centre for the Health Services
